# Infectious salmon anaemia virus infection of Atlantic salmon gill epithelial cells

**DOI:** 10.1186/1743-422X-10-5

**Published:** 2013-01-02

**Authors:** Simon Chioma Weli, Maria Aamelfot, Ole Bendik Dale, Erling Olaf Koppang, Knut Falk

**Affiliations:** 1 Norwegian Veterinary Institute, Ullevålsveien 68, PO Box 750 Sentrum, Oslo, N-0106, Norway; 2 Norwegian School of Veterinary Science, PO Box 8146, Dep, N-0033, Oslo, Norway

**Keywords:** ISAV, Cell tropism, Sialic acid receptor, Gill epithelial cells

## Abstract

Infectious salmon anaemia virus (ISAV), a member of the *Orthomyxoviridae* family, infects and causes disease in farmed Atlantic salmon (*Salmo salar* L.). Previous studies have shown Atlantic salmon endothelial cells to be the primary targets of ISAV infection. However, it is not known if cells other than endothelial cells play a role in ISAV tropism. To further assess cell tropism, we examined ISAV infection of Atlantic salmon gill epithelial cells *in vivo* and *in vitro*. We demonstrated the susceptibility of epithelial cells to ISAV infection. On comparison of primary gill epithelial cell cultures with ISAV permissive fish cell cultures, we found the virus yield in primary gill epithelial cells to be comparable with that of salmon head kidney (SHK)-1 cells, but lower than TO or Atlantic salmon kidney (ASK)-II cells. Light and transmission electron microscopy (TEM) revealed that the primary gill cells possessed characteristics consistent with epithelial cells. Virus histochemistry showed that gill epithelial cells expressed 4-*O*-acetylated sialic acid which is recognized as the ISAV receptor. To the best of our knowledge, this is the first demonstration of ISAV infection in Atlantic salmon primary gill epithelial cells. This study thus broadens our understanding of cell tropism and transmission of ISAV in Atlantic salmon.

## Background

Infectious salmon anaemia (ISA) in Atlantic salmon *(Salmo salar* L*.)* is a World Organisation for Animal Health (OIE) notifiable disease. Clinical ISA is characterized by circulatory disturbances including anaemia, gill pallor, ascites, intestinal congestion, liver and spleen enlargement and petechial haemorrhage of the skin and visceral organs [1]. The disease was first recognized in Norway in 1984, but has been subsequently identified, with high associated mortalities, in farmed Atlantic salmon in Europe, and North- and South America [2-7].

ISA is caused by the infectious Salmon Anaemia Virus (ISAV), the only member of the genus *Isavirus* in the family *Orthomyxoviridae.* The virus has been characterized morphologically [8-11], bio-physiochemically [9,12-15] and genomically [16-23]. However, the pathogenesis of ISAV is not clear and in particular, the site/s of entry into Atlantic salmon remains unknown. In cell culture, ISAV replication has been demonstrated only in SHK-1, ASK-II, TO and Atlantic salmon (AS) cells, although replication of some Canadian ISAV isolates has also been demonstrated in Chinook salmon embryo (CHSE)-214 cells [24-27]. With the exception of AS and CHSE-214, all these cell-cultures are derived from Atlantic salmon adherent head kidney macrophages and mitogen-stimulated peripheral blood leucocytes [24,25,27], and will not, therefore, reveal the virus entry-site into the Atlantic salmon.

The gills, comprising the vasculature and surrounding epithelia [28,29], provide a key interface between the fish and the environment, acting as site for gas exchange, body fluid regulation and waste excretion [30]. As they filter large amounts of water they offer a suitable target for invading infectious agents. While RT-PCR detection of ISAV on whole gill preparations has suggested the gills as an entry-site for ISAV infection [31], *in vivo* studies have only reported ISAV infection within endothelial cells and leucocytes [11,32,33]. We showed previously that gill and hind-gut mucosal epithelial cells express the ISAV 4-*O-*acetylated sialic acid receptor on the cell surface [33], indicating the potential for ISAV-infection of epithelial cells. High prevalences of ISAV positive gills by RT-PCR, have been documented, both for low pathogenic ISAV-HPR0 cases in the Faroe Islands [34], and in high pathogenic ISA outbreaks in Norway [35]. However, analysis of whole gill samples, comprising different cell types, does not allow identification of the specific cell type infected with the virus, or prove the gill to be the entry-site for infection.

If the gill does represent an entry site for ISAV, the virus must penetrate the protective mucosal epithelial barrier. Possible mechanisms include transcytosis, infection of tissue macrophages orchestrated by antigen sampling cells (*i.e.* dendritic cells) or by direct infection of the epithelial cells [36]. Interestingly, mammalian and avian orthomyxoviruses use upper and lower respiratory epithelial cells (analogous to gill epithelial cells) for replication [37,38].

Different approaches have been used to study viral cell tropism. Some studies have used immunohistochemistry [39] or *in situ* hybridization during natural or experimental infection. Others have used *ex-vivo* organ cultures [40,41] or specialized, immortalized cell lines. The study of viral disease in Atlantic salmon is made difficult by the limited availability of specific cell cultures. Thus, in such cases, utilisation of primary cell cultures closely representing the *in vivo* situation can be rewarding [42,43].

In the present study we examined ISAV infection of gill epithelial cells following emersion (bath) challenge and by infection of primary gill epithelial cell cultures. We demonstrated the susceptibility of Atlantic salmon gill epithelial cells to ISAV both *in vivo* and *in vitro*. When compared with ISAV- permissive fish cell lines, primary epithelial cells showed virus yields comparable to SHK-1 cells.

## Results and discussion

Previous studies have demonstrated ISAV receptor expression on gill mucosal epithelial cells [33] and high prevalences of ISAV positive gills by RT-PCR [31,34], suggesting ISAV infection of gill cells. However, such infections have not been confirmed at the cellular level. In the present study, we performed *in vivo* immersion challenge experiments to investigate ISAV infection and cell tropism in Atlantic salmon gills and *in vitro* experiments utilising primary gill epithelial cells.

### ISAV infection of gill epithelial cells following immersion challenge

**Figure 1:**
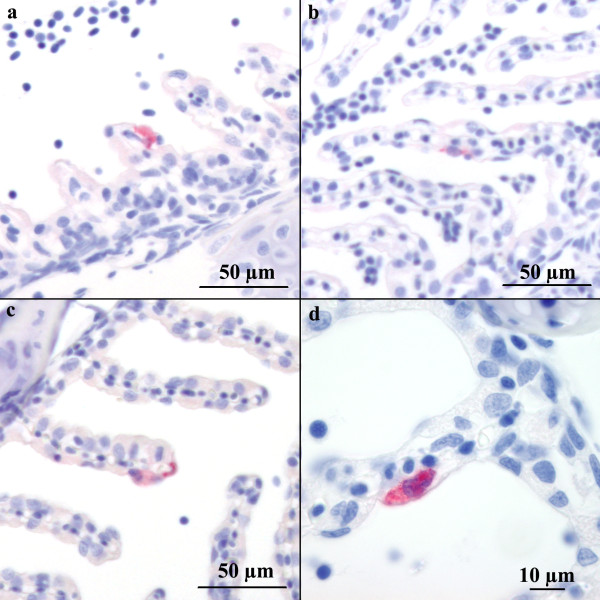
**Immunohistochemical detection of ISAV in formalin fixed paraffin embedded tissue sections from four experimentally immersion challenged Atlantic salmon.** Positive epithelial cells are red.

Previously, we identified, by immunohistochemistry on formalin-fixed paraffin-embedded tissue sections, ISAV infected endothelial cells 8 d.p.i. The ISAV-receptor was localized on gill endothelial and epithelial cells [33]. Only minor pathological changes were observed at autopsy and on histological examination of Atlantic salmon bath challenged with ISAV. Pathological changes included haemorrhage in the skin and eye, sparse ascites, blood stasis in the anterior kidney and minor haemophagocytosis in the spleen. Analysis of gill tissue sections sampled at 8 d.p.i. revealed sparse presence of individual ISAV-positive gill epithelial cells (Figure 1), but not at other time points. Positive endothelial cells were also detected at day 8 p.i., but at low levels. The observation of ISAV-positive gill epithelial cells at day 8 p.i, but not at later time points, indicates that ISAV infects gill epithelial cells at an early stage in the infection, and points to the gill as a possible site of entry and primary replication as previously suggested [31]. The low number of positive cells supports the notion that the gill epithelial cell is not the main target cell for ISAV, which is in agreement with our recent finding that endothelial cells are the main targets for ISAV [33]. In addition, it does not exclude the possibility of other important infection routes, and/or that we have not sampled intensively enough to reveal possible rapid and transient epithelial infection.

### Isolation of primary gill cells from juvenile Atlantic salmon

**Figure 2:**
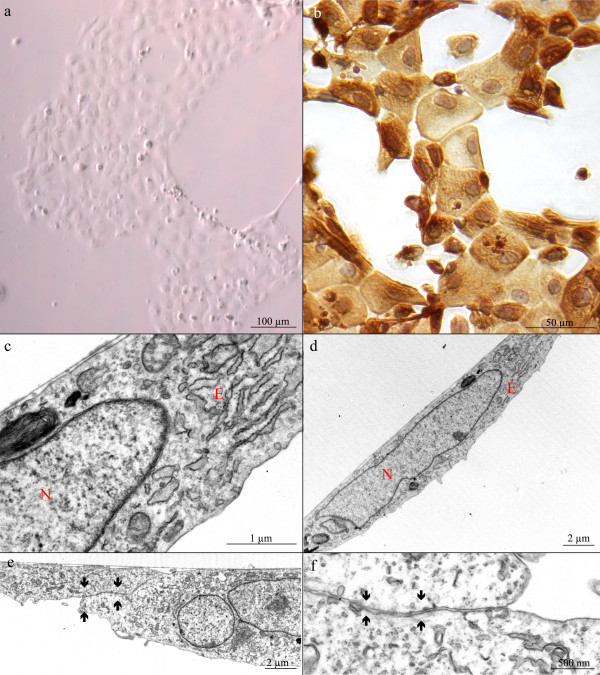
**Atlantic salmon primary gill epithelial cells.** (**a**) Photomicrograph of unstained representative field with typical epithelial cell morphology at 72 h post isolation. (**b**) Anti-cytokeratin immunostaining (AE1/AE3) 72 h post isolation. (**c-f**) Transmission electron microscopy micrographs of monolayer with large nuclei (N), cytoplasmic organelles including endoplasmic reticulum (**E**) and tight junctions (arrows). **c** is a magnification of **d**.

To further explore ISAV infection of gill epithelial cells and to corroborate data from *in vivo* experiments, we carried out a series of *in vitro* experiments using primary gill epithelial cells isolated from juvenile Atlantic salmon. Culture morphology was assessed at varying incubation time points by phase contrast microscopy and immunofluorescence techniques. At 24 h, small colonies of cells had established contact with the substrate, forming tight clusters of 10 to 60 cells. By 48 to 72 h when culture medium was changed and unattached cells removed, the colonies had expanded 2-fold (Figure 2a). Cells spread faster and projected upwards from the substrate when seeded abundantly (4.0 × 10^5^ cells/cm^2^), but when seeded more sparsely (2.0 × 10^5^ cells/cm^2^), they appeared flattened and the time taken to form a complete monolayer was extended. Viability of cells was demonstrated by trypan-blue exclusion [44] of more than 90% of the cells at day 7 post isolation, indicating prolonged viability (data not shown).

### Epithelial origin of isolated primary gill cells

To confirm that the isolated primary gill cells were of epithelial origin, immunocytochemical staining with an epithelial-specific cytokeratin marker was performed. Cells consistently stained positive for cytokeratin, and showed little contamination with other cell types (Figure 2b), indicating that the cultured gill epithelial cells were largely monomorphic. Using transmission electron microscopy (TEM), we assessed 7-day-old cultures for epithelial characteristics. We found tight junctions, nuclei and cytoplasmic organelles including endoplasmic reticulum (Figure 2c-f), all features consistent with epithelial cells. We were however, unable to localize desmosomes. A possible explanation for this may lie in our sample preparation, as some studies indicate that a freeze fracture technique is required to characterize complete regions of tight membrane-to-membrane contact [45].

### Detection of ISAV 4-*O-*acetylated sialic acid receptor on primary gill epithelial cells

**Figure 3:**
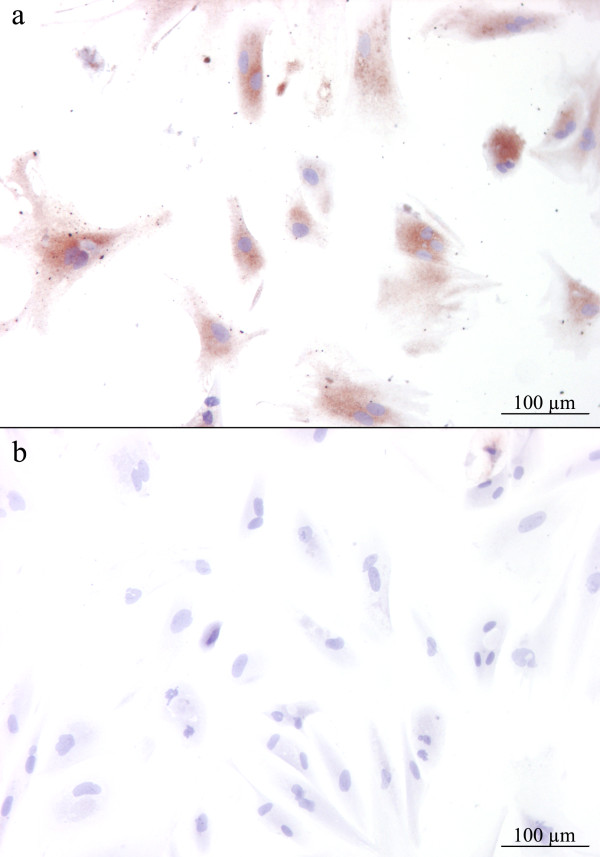
**Detection of virus receptor using virus histochemistry with Envision® system and 3,3’-diaminobenzidine as substrate which gives a brown colour at the binding site in Atlantic salmon primary gill epithelial cells incubated at room temperature for 1 h with ISA viral antigen (a).** Negative control cells (**b**) NaOH treated (*i.e.* saponification) prior to incubation with ISA viral antigen.

Species preference and tissue tropism are critical for ISAV transmission and infection. As documented previously, ISAV binds 4-*O*-acetylated sialic acid receptors. Recent evidence gathered by virus histochemistry on Atlantic salmon tissue using ISAV antigen as a probe, indicated that 4-*O*-acetylated sialic acid receptors are expressed on the surface of host cells; including endothelial cells as well as gill- and hind gut mucosal epithelial cells [15,33]. To assess the potential infection- and tissue tropism of ISAV for gill epithelial cells, we investigated the presence/absence of sialic acid receptors on non-infected primary gill epithelial cell isolates by virus histochemistry. Expression of the ISAV receptor on gill epithelial cells was confirmed (Figure 3a), suggesting that ISAV may have the potential to infect and replicate in gill epithelial cells. The primary epithelial cells were washed extensively to remove serum and other culture media components prior to incubation with antigen. However, some of the positive staining might be due to non-specific binding of antigen to cell surface-associated components rather than to integral membrane glycoproteins. To further clarify this we performed control experiments that included exposing the gill epithelial cells to a mild alkaline solution; a well-established method for de-*O-*acetylation of sialic acids [46]. This treatment completely abolished the staining, confirming the specificity of the reaction (Figure 3b).

### ISAV infection of primary gill epithelial cells

**Figure 4:**
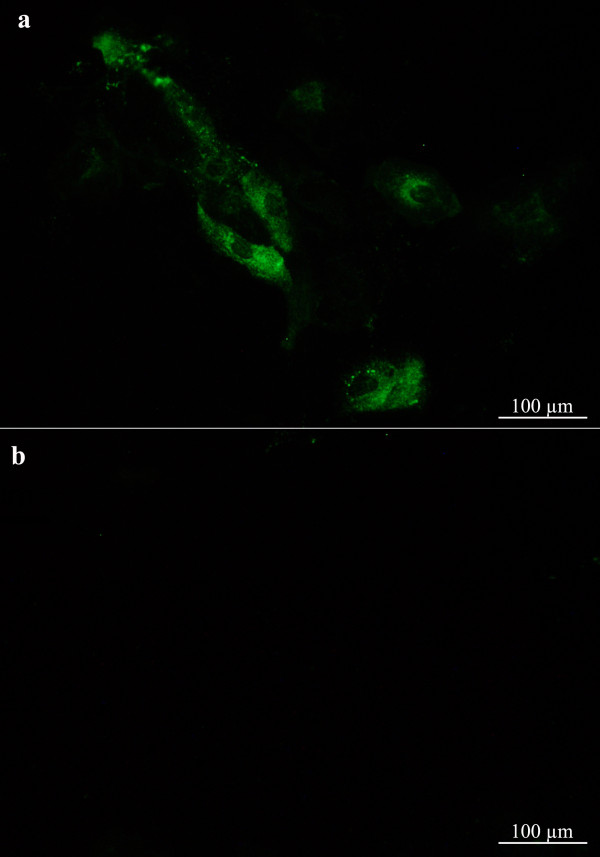
**Localisation of ISAV in Atlantic salmon primary gill epithelial cells.** Infected cells (**a**) fixed at 4 day p.i. and subjected to immunofluorescence (IFAT) staining (green fluorescent staining) with mouse monoclonal antibody directed against the ISAV nucleoprotein. No viral antigen was detected in mock-infected cells (**b**).

We have demonstrated ISAV infection of epithelial cells following *in vivo* immersion challenge (Figure 1). We have also shown that primary gill cell cultures possess epithelial characteristics (Figure 2) and express 4-*O-*acetylated sialic acid receptors (Figure 3), which correlates with previous reports on 4-*O-*acetylated sialic acid expression [15,33]. In order to explore possible ISAV-gill epithelial cell tropism, we performed *in vitro* ISAV infection of gill epithelial cells. We found that ISAV readily infected epithelial cells as shown by IFAT (Figure 4a). No viral antigen was detected in mock-infected cells (Figure 4b). The positive staining and receptor binding are consistent with previous reports from endothelial cells [13,33]. To the best of our knowledge, this is the first report demonstrating ISAV infection in epithelial cells and provides new insight into *in vivo* ISAV replication in epithelial cells.

### ISAV replication assay

As the results from *in vivo* experimental infection correlated with *in vitro* infection, we compared viral particle production in primary gill epithelial cells with ASK-II, SHK-1 and TO cells. ASK-II, SHK-1 and TO cells are commonly used for both research and routine diagnostics and are permissive to ISAV [8,25,27]. We found virus yields in gill epithelial cells comparable to SHK-1 cells, 10-fold lower than TO cells and 100-fold lower than ASK-II cells (Table 1). This study demonstrates not only the capacity of ISAV to replicate in primary gill epithelial cells, but also that virus produced in the primary epithelial cells replicated in ASK-II cells that was used for virus titration. The virus yield obtained in the present study from primary gill cultures is clearly lower than that determined on ISAV infected SHK-1 (10^6^ to 10^7^) [9] and TO (10^7.8^ to 10^9.1^) [27] cells. However, virus supernatants in our study were harvested on day 4 p.i., when rounding of cells was clearly observed. The difference in titres may be due to timing, as virus titres for SHK-1 [9] and TO cells [27] were determined on day 8 and 12 p.i. respectively. Although virus titres were lower, we have documented that primary epithelial cell cultures have the potential to be used for study of ISA pathogenesis and ISAV transmission mechanisms.

**Table 1:** Differential permissiveness of Atlantic salmon primary gill epithelial cells, ASK-II, SHK-1, and TO cells to ISAV infection

**Cells**	**†ISA virus titres (TCID**_**50**_**/mL)**
Atlantic salmon primary gill epithelial cells	1.0 x 10^5^
ASK-II	1.0 x 10^7^
SHK-1	4.0 x 10^5^
TO	1.0 x 10^6^

Note: † Titres at 4 days p.i.

## Conclusion

In summary, this study constitutes the first documentation of ISAV infection of gill epithelial cells, *in vivo* and *in vitro*, and provides evidence of the gill as a potential port of entry for ISAV. The Atlantic salmon primary gill epithelial cells described here were used to study ISAV cell tropism and infection. The cells are not a new cell line, and not optimal for maximum yield of pathogenic ISAV strains. Also, attempts to passage the cells were unsuccessful. However, they are of interest for two reasons. Firstly, they possess epithelial characteristics and express ISAV 4-*O-*acetylated sialic acids receptors critical for ISAV infection. Secondly, they provide further evidence that Atlantic salmon gill epithelial cells could be a target for ISAV. They may also provide a system for working with so far uncultivable gill-infecting agents, *e.g.*, low pathogenic ISAV HPRO strains, and also other suspected gill-pathogenic agents such as pox virus [47].

## Materials and methods

### Preparation of primary gill epithelial cells

Primary cultures of gill epithelial cells from juvenile Atlantic salmon (5-10 g) were isolated as previously described [48], with some modifications. Briefly, fish were euthanized by anaesthetic overdose in ethyl 3-aminobenzoate methanesulfonate salt (Sigma-Aldrich), gill arches excised, washed in phosphate buffer saline (PBS) without Ca_2_^+^ and Mg_2_^+^ containing 200 μg/mL Penicillin/Streptomycin (Invitrogen), 400 μg/mL gentamicin sulfate (Lonza), and 2.5 μg/mL Amphotericin B (Lonza). Cultures were prepared from filaments utilising three cycles of enzymatic digestion with trypsin-EDTA (Lonza), and cell washing in PBS supplemented with 2% foetal bovine serum (FBS). Cells were re-suspended in L-15 medium supplemented with FBS (20%), Penicillin/Streptomycin (100 μg/mL), ß-mercaptoethanol (1 mM), nonessential amino acid (0.1 mM), L-glutamine (2 mM) and seeded on 24 well plates at 15°C. To monitor growth characteristics, cells were observed daily by light microscopy and assessed for viability by trypan-blue exclusion [44], morphology and susceptibility to ISAV infection.

### Virus

The highly pathogenic Norwegian ISAV strain Glesvaer/2/90 [8] was propagated in ASK-II cells [25] in Leibovitz L-15 medium (L-15) supplemented with 10% FBS, glutamine (4 mM), and gentamicin (50 μg/ml) at 20°C. The virus was used for *in vivo* (fish immersion experiments), for *in vitro* infection of the primary gill epithelial cells, and for preparation of ISAV antigen for virus histochemistry as previously described [33]. Unless otherwise stated, primary gill epithelial cells with the fluid overlay removed were inoculated with virus at a multiplicity of infection of 0.1 in L-15 with 2% FBS. Infectivity titrations were done by end point titration in 96-well culture plates as previously described [9].

### Experimental infection and fish sampling

The infection experiment was performed at the Norwegian Veterinary Institute in freshwater tanks at 8°C. A total of 31 Atlantic salmon presmolts (20 g) confirmed free of ISAV, infectious pancreatic necrosis (IPNV), proliferative kidney disease (PKD) and Salmonid alpha virus (SAV) by RT-PCR analysis were used. Fish were challenged by immersion (bath) for 3 hours with 10^4^ TCID_50_ per ml. Un-infected control fish were kept in a separate tank. Three - five fish were sampled at 8 h, 24 h, 48 h, 8 days and 20 days post infection (p.i.), and gill tissues collected in buffered 4% formaldehyde. Fish were anesthetized with methane tricaine sulphonate (MS222, Sigma, 0.1 mg/mL) before handling.

### Ethical considerations

Fish sampling for preparation of primary gill epithelial cells and experimental infection were preformed according to internationally recognized ethical guidelines. The experiments were approved by The Norwegian Animal Research Authority (NARA); identification number 2697.

### Immunohisto- and cytochemistry

Immunohistochemistry was performed as previously described [49] on gill samples from *in vivo* immersion experiments and tissues samples from the Faroe Islands. Briefly, formalin-fixed paraffin-embedded tissue sections were de-waxed and subjected to microwave oven treatment. Rabbit antibody to recombinant ISAV NP [50] and Vectastain ABC-AC kit (Vectastain anti rabbit Ig ABC-AP kit, AK 5001, Vector Laboratories, Inc.) were used for detection, employing Fast Red (1 mg/ml) and Naphtol AS-MX phosphate (0.2 mg/ml) with 1 mM Levamisole in 0.1 M TBS (pH8.2) as substrate.

Immunocytochemistry was performed to demonstrate purity of the primary gill epithelial cultures as previously described [51]. Briefly, cultures were washed in PBS and fixed in 4% formaldehyde in PBS for 10 min. Non-specific binding was blocked by incubation in 5% FBS in PBS for 20 min. Labelling was performed by overnight incubation at 4°C with mouse monoclonal anti-cytokeratin (AE1/AE3) (Invitrogen) diluted 1:50 in PBS with 2% FBS, followed by 30 min incubation with HRP-labelled secondary antibody (Envision® K4007; DAKO). 3,3’-diaminobenzidine (DAB) was used as a substrate.

### Immunofluorescent labelling

Immunofluorescent antibody test (IFAT) was used for ISAV detection in infected primary gill epithelial cells as previously described [13], with some modifications. Briefly, 7 day old primary gill cultures were inoculated with ISAV at a multiplicity of infection (MOI) of 0.1 in Leibovitz's L-15 medium with 2% FBS and allowed to adsorb for 2 h at 15°C. The inoculum was removed and fresh L-15 medium supplemented with FBS (10%), Penicillin/Streptomycin (100 μg/mL), ß-mercaptoethanol (1 mM), nonessential amino acid (0.1 mM), L-glutamine (2 mM) added and incubated for 4 days. Cultures were washed in PBS, fixed in 80% acetone for 10 min, and IFAT was performed with anti ISAV haemagglutinin esterase (HE) monoclonal antibody [13] and FITC-labelled goat anti-mouse Ig (Southern Biotech).

### Transmission electron microscopy

Transmission electron microscopy (TEM) was performed on 7-day-old primary gill epithelial cells as previously described [52]. Briefly, cultures were washed in PBS, fixed in 2.5% glutaraldehyde in 0.5 M cacodylate buffer postfixed in 2% OsO4 in 0.1 m cacodylate buffer pH7.2 for 2 h, dehydrated in ascending concentrations of ethanol and embedded in LX 112 Resin (Ladd Research Industries). Ultra-thin sections were mounted on uncoated copper grids, stained with uranyl acetate and lead citrate using standard methodology before examination.

### Virus histochemistry

For detection of the ISAV 4-*O*-acetylated sialic acid receptor, virus histochemistry was performed on primary gill cultures as previously described [33]. Briefly, cultures were washed in PBS, fixed in 4% formaldehyde in PBS for 10 min. Labelling was performed by incubating with each of the following for 1 h: ISAV antigen (100 HAU mL-^1^), anti ISAV HE monoclonal antibody [13] and HRP-labelled secondary antibody (Envision® K4007; DAKO). DAB was used as a substrate. Cultures were counterstained with Mayer’s haematoxylin. To document binding specificity, control cells were de-*O*-acetylated by mild alkaline saponification using 0.1 M sodium hydroxide for 30 min [46].

### ISAV replication assay

To further characterize ISAV infection of the primary gill epithelial cells, comparisons of ISAV replication in primary gill epithelial cells were made with ASK-II, SHK-1 and TO cells. Cultures of primary gill epithelial cells, ASK-II, SHK-1 and TO cells were inoculated with ISAV as previously described, and incubated for 4 days. Supernatants were collected four days p.i., and infectivity titrations performed on ASK-II cells by end point titration in 96-well culture plates as previously described [9]. The 50% tissue culture infective dose (TCID_50_) was estimated by the method of Kärber [53].

## Competing interests

The authors declare that they have no competing interests.

## Authors’ contributions

SCW and MA carried out the experiments and drafted the manuscript. KF participated in design and coordination of the experiment, and aided drafting of the manuscript. OBD and EOK participated in coordination and helped to draft the manuscript. All authors read and approved the final manuscript.
